# Investigating the effective temporal resolution in a task-based functional MRI experiment at 7 T MRI using a dynamic phantom

**DOI:** 10.1162/imag_a_00309

**Published:** 2024-10-10

**Authors:** Guy Baz, Rita Schmidt

**Affiliations:** Weizmann Institute of Science, Department of Brain Sciences, Rehovot, Israel; The Azrieli National Institute for Human Brain Imaging and Research, Weizmann Institute of Science, Rehovot, Israel

**Keywords:** temporal resolution, functional MRI, ultra-high field MRI, multi-echo

## Abstract

An increasing number of human fMRI studies aim to discern the time delays between evoked responses under different stimuli conditions in different brain regions. To achieve that, a primary goal is to acquire fMRI data with high sampling rates. This task is now possible with ultra-high field (≥7 T) MRI and the advancement of imaging acceleration methods. Consequently, it becomes imperative to understand what is the actual or effective temporal resolution (ETR) that is realized in given settings of an fMRI experiment. In this study, we utilized a dynamic phantom to reliably repeat a set of scans, generating a “ground truth” signal with controllable onset delays mimicking fMRI responses in a task-based block-designed fMRI. Here, we define the ETR and quantify a scan’s ETR using the dynamic phantom. The quantification was performed for various scanning parameters, including echo time (TE), repetition time (TR), voxel size, and contrast-to-noise ratio (CNR). We further show that combining data from multi-echo EPI can improve the ETR (i.e., reduce it). In addition, parameters of the fMRI paradigm were examined, including the blocks’ length and density. As tissue properties (e.g., level of iron deposition) affect the CNR and thus change the ETR, we examined the signal rise mimicking not only the cortex, but also the basal ganglia (known for its high iron deposition). Combining multi-echo data, the estimated ETR for the examined scans was 151 ms for a cortex-mimicking setup and 248 ms for a basal ganglia-mimicking setup, when scanning with a sampling time (i.e., TR) of 600 ms. Yet, a substantial penalty was paid when the CNR was low, in which case the ETR was even larger than the TR. A feasibility set of experiments was also designed to evaluate how the ETR is affected by physiological signal fluctuations and the variability of the hemodynamic response. This study shows the viability of studying time responses with fMRI, by demonstrating that a very short ETR can be achieved. However, it also emphasizes the need to examine the attainable ETR for a particular experiment.

## 1 Introduction

Since its discovery at the beginning of the 90s (Ogawa et al., 1990), functional magnetic resonance imaging (fMRI) has gained increasing popularity in the study of human brain function. This is attributed to its remarkable ability to image whole-brain activity at a much higher*spatial*resolution than other tools, such as electroencephalography (Rosen & Savoy, 2012;Stelzer et al., 2014). The*temporal*capabilities of fMRI, on the other hand, are still unclear.

It is well known that the hemodynamic response—reflected in the signal changes measured in MRI—acts as a temporal filter (S.-G. Kim et al., 1997). Consequently, the signal’s rise time is relatively slow (4–6 seconds) compared to the neural activity it seeks to measure. This drawback has prompted efforts to evaluate fMRI’s temporal responses using deconvolution approaches (Chai et al., 2019;I. Kim et al., 2024). Others have explored methods to directly probe the timing-events across trials. These include examining onset delay (Menon et al., 1998;Setzer et al., 2022), the temporal order of activity across brain regions or experimental conditions (Lin et al., 2013), or the decoding of stimuli with different time lags from voxel data (Misaki et al., 2013;Vu et al., 2016;Wittkuhn & Schuck, 2021). Their results indicated that time delays as fast as 100 ms can be captured. On the other hand, recent studies on oscillatory activity with fast fMRI detected cycles down to a 1-second period (Lewis et al., 2016).

While it is common to refer to the repetition time (TR) as the temporal resolution of the scan, several refined techniques were also introduced to capture much faster temporal changes than the time required to acquire an image. Among them are the jittering approach (Watanabe et al., 2013), an inverse reconstruction that can estimate fMRI-related changes with a 100 ms sampling rate (Lin et al., 2008); line scanning, which demonstrates an up to 50 ms sampling rate (Nunes et al., 2021;Raimondo et al., 2021;Yu et al., 2014); and reordering of the phase encoding lines, which can reach an up to 5 ms sampling rate (Silva & Koretsky, 2002;Toi et al., 2022).

At the same time, technological improvements in recent years have boosted the fMRI sampling rate and spatial resolution for the commonly used fMRI pulse sequence—the Echo-Planar Imaging (EPI). Most notably, in-plane and simultaneous multi-slice acceleration methods now allow a much faster whole-brain image acquisition, reaching a >1 Hz sampling rate (Chen et al., 2015). In addition, ultra-high field 7 T MRI supplies an enhanced SNR, which allows further acceleration (Lewis et al., 2016).

While improving the methodology and refining techniques for increasing sampling rates, it is imperative to understand what is the actual or effective temporal resolution (ETR) that can be achieved in defined settings of an fMRI experiment. Shortening the TR and thus increasing the sampling rate does not necessarily ensure the shortest ETR. Specifically, a scan’s SNR and contrast-to-noise ratio (CNR) play a key role in determining the effective temporal resolution (ETR) of a scan (Bandettini, 2000;Murphy et al., 2007). However, investigating the ETR of fMRI using real subjects’ data is especially challenging due to the high variability of the neurovascular response, both across subjects and within the same subject. Thus, to accurately assess the ETR of a certain scan, knowledge regarding the expected response is crucial. Recently, the ability to do so, that is, to generate a “ground-truth” signal, was established through the introduction of a*dynamic phantom*(DeDora et al., 2016). It was shown that using such a phantom, one can accurately assess the scan’s inherent CNR while imitating the expected signal of a resting-state scan (Kumar et al., 2021).

In this study, we utilized the dynamic phantom to reliably repeat a set of scans, generating signals with controllable onsets that mimic*in-vivo*-evoked responses in a task-based block-designed fMRI. Following that, we defined and quantified the ETR and its dependence. The goal of this study is not to measure the time delay between two evoked signals, but to investigate what is the time resolution that a chosen set of scan parameters and an fMRI paradigm can supply. The time resolution here represents the minimal time delay that can be discerned between evoked responses with high enough statistical confidence. Shortening the ETR can be achieved by different scanning strategies. We can use the slow hemodynamic response to our advantage, acquiring enough time points during the signal rise and fall to capture the differences between the evoked responses. In this case, high SNR measurements will uncover the differences in magnitude between two evoked signals even with a very small time delay, while a low SNR will bury the signal differences in noise (see example in Supporting InformationFig. S1). Therefore, although a restricted field-of-view can achieve a shorter TR, it also reduces the SNR, which prolongs the ETR. Increasing the scan duration, number of trials, or number of subjects can improve the statistical power and thus shorten the ETR. Here, the effect of the scan parameters is investigated, including TE, TR, and voxel size, along with the resulting CNR. In addition, multi-echo EPI was previously demonstrated to improve the fMRI statistics (Gonzalez-Castillo et al., 2016). In this study, we examine its ability to shorten the ETR. We also test the effect of the stimuli design and of the expected response on the ETR, including variation in the response shape and temporal variations (due to a simple breathing model).

The ETR can also differ among brain regions. The SNR in the sub-cortex is lower, implying that the ETR in those regions is hampered as well. In the basal ganglia (BG), the high iron concentration leads to especially short T_2_^*^values, which, in turn, generate a lower signal. A potential method for overcoming the lost signal is to use multi-echo EPI (Kundu et al., 2017;Puckett et al., 2018), offering better detection of the signal change across a dynamic range of T_2_^*^values. Here, we investigate the ETR in short- and long-T_2_^*^setups to mimic the characteristic properties of the cortex and BG, respectively, and to study how multi-echo EPI can improve the ETR.

## 2 Methods

### 2.1 The dynamic phantom

**Fig. 1.**
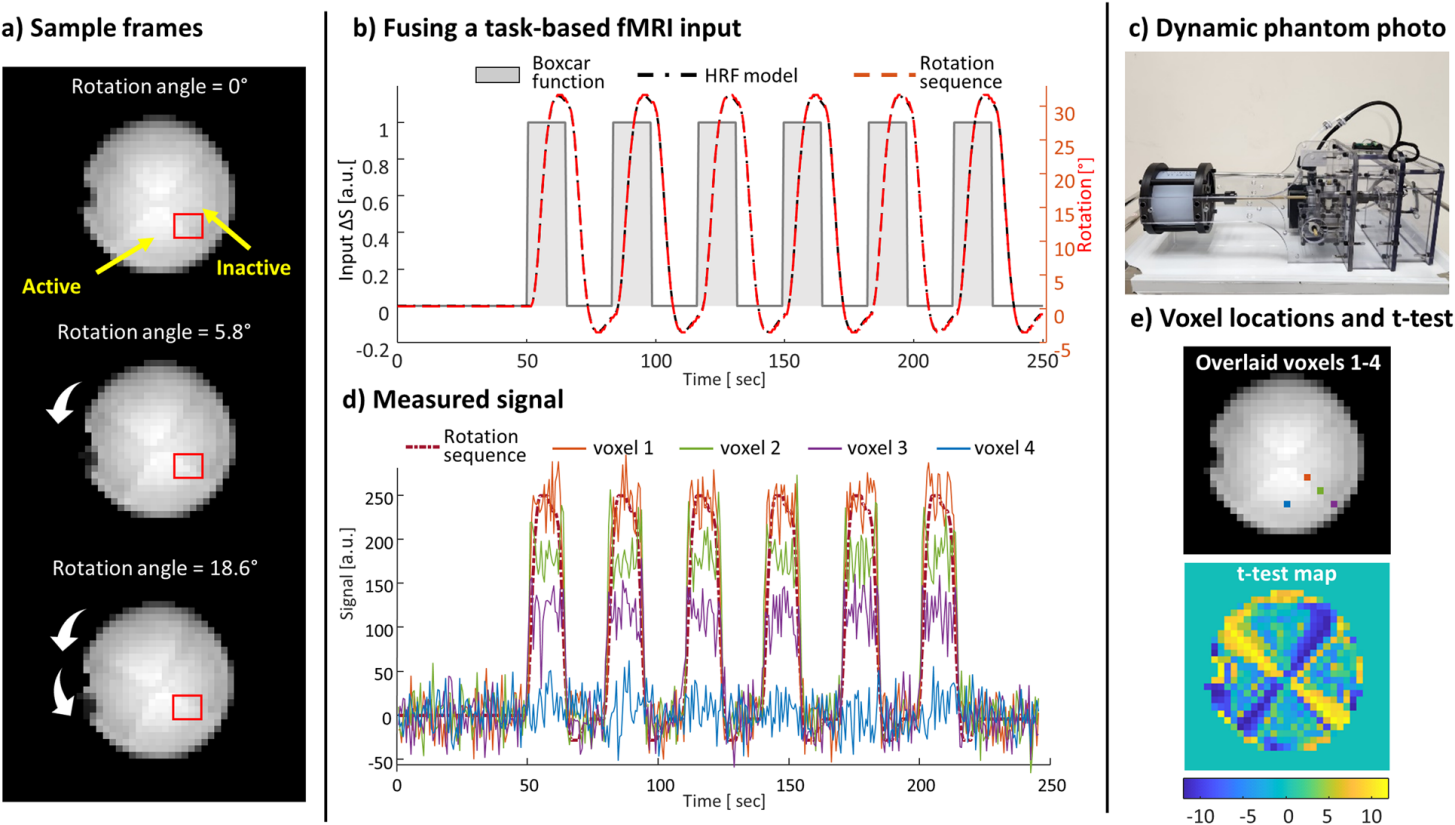
Using a dynamic phantom to create an “fMRI-like” signal. (a) Sample frames of the MRI images of the phantom capturing three rotation angles. The agarose-head’s four quarters, two containing a high agarose concentration (representing the signal in the “Inactive” state), and two a low agarose concentration (representing the signal in the “Active” state). The region of interest shown in red demonstrates the signal captured at three different rotation angles in the same voxel. (b) Fusing a task-based fMRI input. An HRF model response is calculated for a boxcar function of a task-based paradigm. A corresponding rotation sequence for the BrainDancer is generated. (c) BrainDancer phantom photo. (d) Measured signal for a sample of voxels shown in (e). (e) An MRI image with the overlaid voxel locations and corresponding t-test map that shows regions sensitive to the phantom rotation.

The dynamic phantom (“Brain dancer”, ALA scientific Inc., New York, USA) description and its principle of operation can be found in (Kumar et al., 2021). The phantom comprises two agarose compartments. The first represents an “active” state, having a lower agarose concentration and thus higher T_2_* values. The second represents an “inactive” state, having a higher agarose concentration and thus lower T_2_* values. The deviation between the agarose concentration in the compartments results in a ~5% deviation in the T_2_*. To generate dynamic signal changes, the cylindrical phantom rotates about its axis, which changes the T_2_* properties in a specific voxel in every captured scan. The rotation of the cylindrical head is set by a controller box that receives as an input a sequence of clockwise and counterclockwise rotation angles. The execution is activated by a trigger set in the MRI scan, providing synchronization between the rotation sequence and the MRI scan.Figure 1shows a sample of MRI scans during the phantom rotation, the rotation sequence, and the measured signal changes in sampled voxels. The signal from a given voxel depends on the ratio of the two T_2_^*^compartments within the voxel. The signal rise in imaging voxels varies due to the rate at which this ratio changes when the phantom rotates, depending on the voxel location (seeFig. 1). This provides a range of signal rises in a single slice. Multi-slice acquisitions provide multiple voxels with the same signal behavior within a single scan, while the thermal noise is determined by the scan parameters.

### 2.2 Design of a mimicked task-based fMRI signal

The dynamic signal was designed to mimic a block-design paradigm through a convolution of the hemodynamic response function (HRF; using the*spm_hrf*function of the SPM12 package (“SPM Toolkit,” 1997)) with a stimulus boxcar function. A rotation sequence that mimics an evoked signal change for a block-designed fMRI was defined (Fig. 1b). The paradigm was set as time blocks of 12 seconds “ON” and 18 seconds “OFF” (if not stated otherwise). In addition, the rotation sequences were generated with different predefined time-shifts of the “ON” onset time (Δt). The rotation angles were set inside the phantom’s rotation limits, that is, a rotation angle range of 0.439° to 3.512° in steps of 0.0439°. Therefore, the angles were rounded to the closest value, if necessary. At the beginning of each sequence, 60 seconds without rotation was added for baseline estimation.

### 2.3 Design of a phantom with short T _2_^*^ to mimic the properties of the BG

**Fig. 2.**
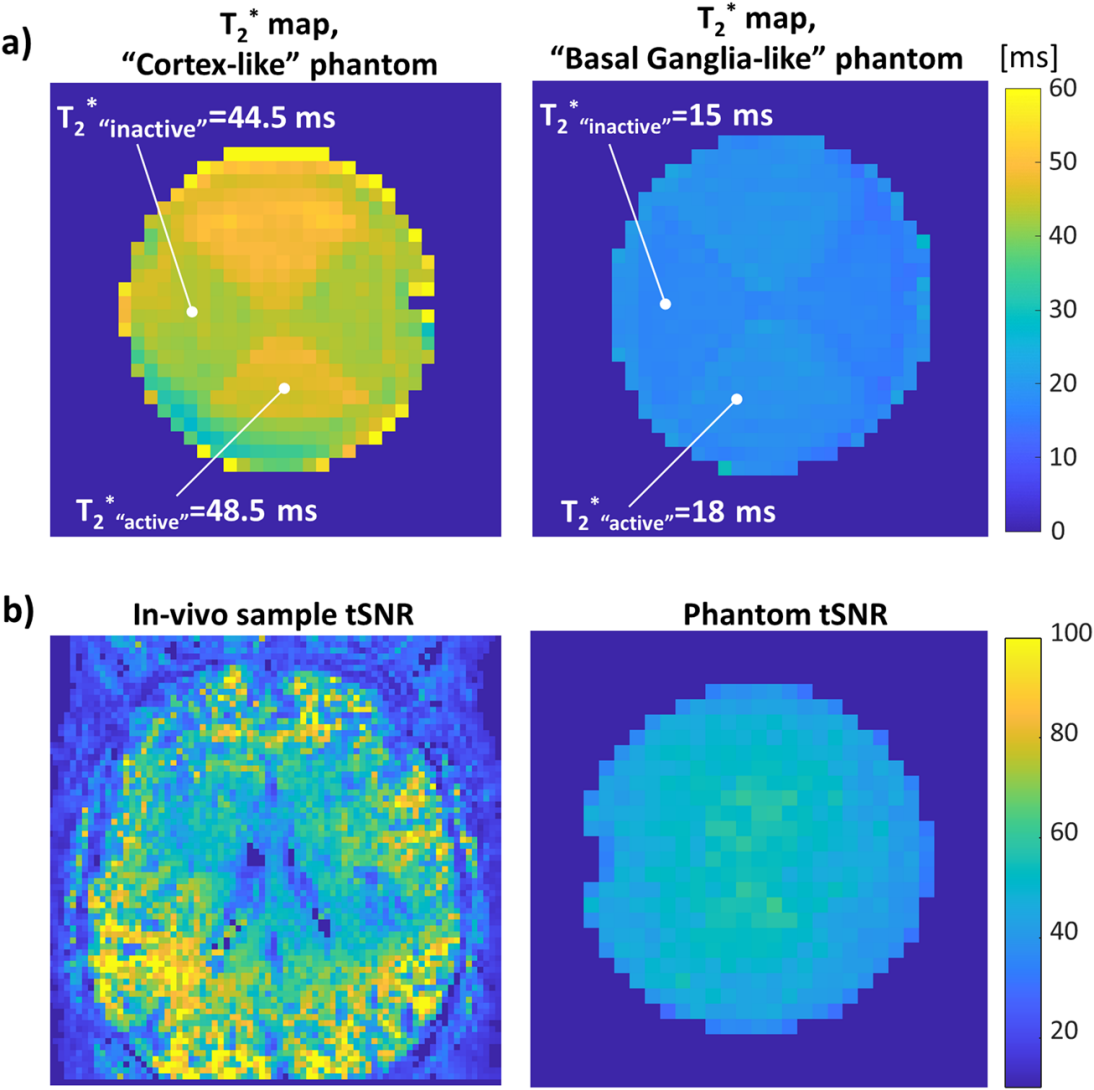
Mimicking brain tissue and tSNR properties. (a) T_2_* maps of two phantom configurations: on the left, a phantom that mimics the “cortex-like” properties; on the right, a “basal-ganglia-like” configuration. (b) tSNR comparison of a human scan and a phantom scan for a multi-echo dataset.

In addition to the vendor’s agarose composition, an in-house composition with higher agarose concentrations was prepared to mimic a shorter T_2_^*^that better represents the BG regions. The vendor’s composition had T_2_^*^values of ~44.5 ms and ~48.5 ms for the “inactive” and “active”, respectively, which was termed “long-T_2_^*^phantom”. The in-house composition had T_2_^*^values of ~15 ms and ~18 ms, respectively, and thus was termed “short- T_2_^*^phantom”. The T_2_^*^was measured using multi-echo GRE at 7 T MRI. The two compositions were used to examine the difference in ETR in tissues with short and long T_2_^*^.Figure 2apresents T_2_^*^maps of the tested phantoms.

### 2.4 Definition and quantification of the effective temporal resolution (ETR)

**Fig. 3.**
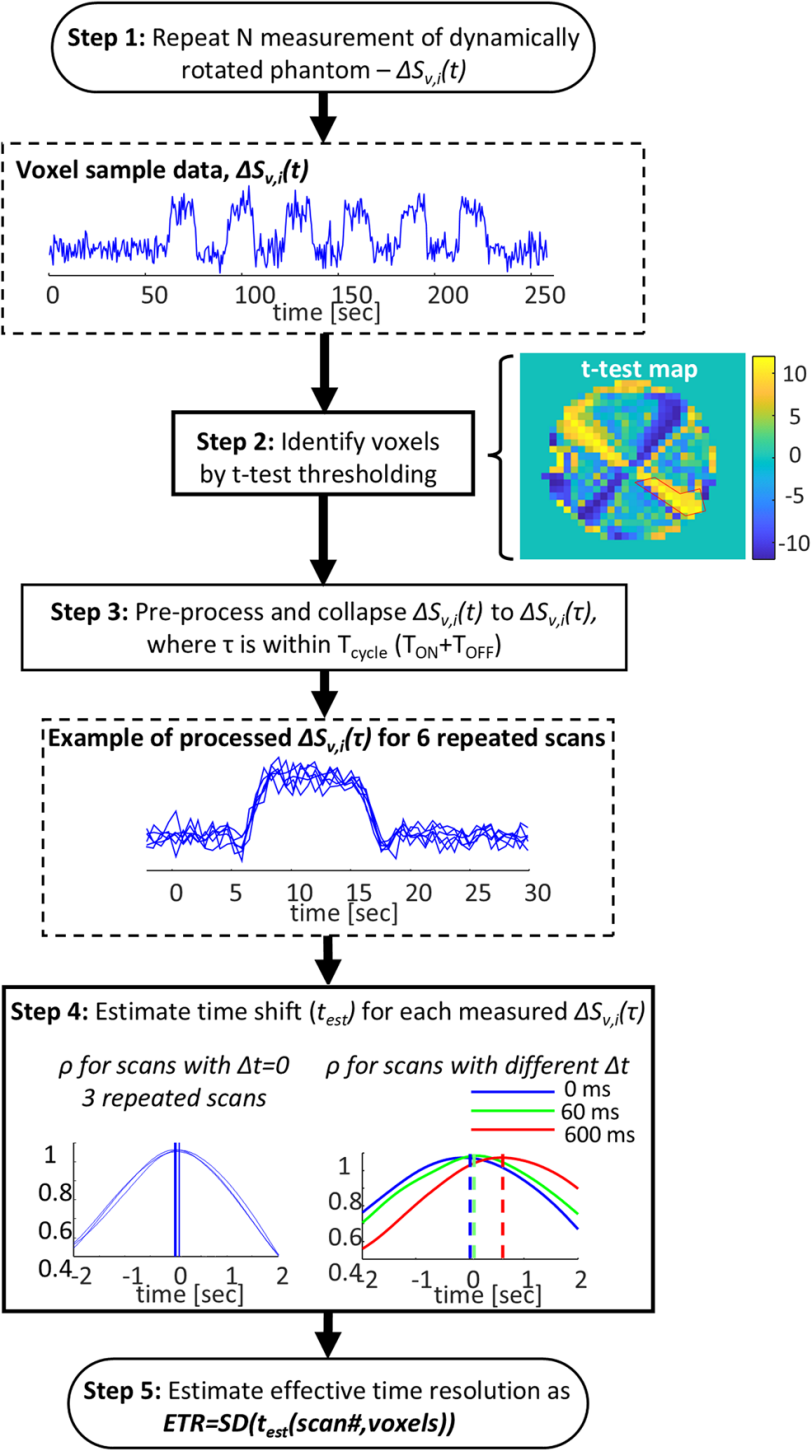
Schematic overview of the effective temporal resolution (ETR) quantification/estimation.

The quantification of the ETR comprised 5 steps (Fig. 3).

***Step 1.***A set of consecutive repeated scans with the same parameters was performed, and the signal percent change (ΔS) of each voxel (*v*) per scan (*i*) was calculated:

$$\Delta S_{v,i}\left(t\right)=100\;\cdot \;\frac{S_{v,i}\left(t\right)-{\langle S_{v,i}\left(t\right)\rangle }_{t}}{{\langle S_{v,i}\left(t\right)\rangle }_{t}}$$,

where${\langle S_{i,v}\left(t\right)\rangle }_{t}$is the mean signal over time of voxel*v*within scan*i*.

***Step 2.***Only voxels that capture the phantom’s rotation (such as voxels 1–3 inFig. 1) were kept, while discarding voxels not sensitive to the rotation (e.g., voxel 4 inFig. 1). For this, a t-test map for the first scan in the set was calculated, comparing the ΔS in each voxel to an expected signal change. Only voxels with a high t-test (t-test > 10 for the long-T_2_^*^phantom and t-test > 8 for the short-T_2_^*^phantom) were included in the following steps of the analysis.

***Step 3.***A collapsed ΔS to a single short, effective${\hat{\Delta S}}_{v,i}\left(\tau \right)$with a duration of one cycle of “ON” and “OFF” states was calculated. For this, the scan time,*t*, was rewritten as t(τ,n) =*t*_0_+*τ *+ n*T*_cycle_, where*t*_0_is an initial time shift (see below),*T*_cycle_the duration of a single pair of “ON” and “OFF” states, and*τ*the time within the*n*-th cycle of duration*T*_cycle_(*n*= 1,2,…,*N*_cycles_), where*N*_cycles_is the total number of cycles in the scan. The value of*t*_0_was set so that for a chosen*δt*(2 seconds here) and τ ∈ [-*δt*,*T*_cycle_—*δt*]; the time*τ*= 0 is the onset of the “ON” state.*δt*is chosen to ensure that we can capture the signal rise. The τ window length should be at most T_cycle,_to ensure no overlap between consecutive response datasets. Using these definitions, the collapsed average signal was defined as

$${\hat{\Delta S}}_{v,i}\left(\tau \right)={\langle \Delta S_{v,i}(\text{t}\;\text{(}\tau ,\text{n)}=\tau \rangle }_{n}$$,

where〈·〉denotes an average over different n. Similarly, a matching standard deviation of the signal change was defined,$\sigma_{\Delta S}=SD_{n}(\Delta S_{v,i}\left(t\left(\tau ,n\right)=\tau \right)$(SDn is the standard deviation over n).

In order to compare different scans, the effective ΔS was further normalized:

$$\begin{array}{l} \Delta S_{v,inorm}\left(\tau \right)=({\hat{\Delta S}}_{v,i}\left(\tau \right)-\min(\Delta S_{OFFwindow}))\\ /\;(\max(\Delta S_{ONwindow})-\min(\Delta S_{OFFwindow})), \end{array}$$

where ΔS_OFFwindow_is ΔS in the “OFF” state, calculated within a window around onset time in a range of -1 to 1 second, and ΔS_ONwindow_is ΔS in the “ON” state. For the T_ON_= 12 seconds, the ON_window_was within a range of 5–15 seconds.

***Step 4.***This step estimated the response time delays per scan, within a set of scans. The time delay was relative to a measurement set as the reference one (chosen from the first scan in the set). In experiments that examined the ETR as a function of repeated stimuli, the reference measurement was defined as one of the measurements within a single cycle. The time delay in the reference was set to zero and was not taken into account in the statistics. For this, all the$\Delta S_{v,inorm}(\tau )$were interpolated (using time steps of TR/20) and the first scan in the set was denoted as a reference scan ($\Delta S_{v,refnorm}(\tau )$). The time delay t_est v,I_– between each scan and the reference was estimated using cross-correlation. Each dataset$\Delta S_{v,inorm}(\tau )$was replicated and shifted in steps of TR/20 within a range of -3 to 3 seconds. A cross-correlation (ρ) between the shifted$\Delta S_{v,inorm}(\tau )$and the reference signal$\Delta S_{v,refnorm}(\tau )$was calculated at each time shift (seeFig. 3). The time shift with the maximal cross-correlation was set as the estimated time delay, t_est v,I_.

***Step 5.***The ETR was evaluated as one standard deviation of the above estimated time delays (which represents 68% of the observed data in a distribution) for all scans and voxels with the same onset: ETR = SD(*t_est v,i_*). Since no time delay is expected, this standard deviation of time delays provides us with an estimate of the experimental*time shift resolution*, that is, the*minimal time shift difference*that can be experimentally discerned. When the scans were also repeated with different onsets, Δt, the final ETR was averaged over the ETRs for each onset.

Scans of multi-echo EPI were used, and the ETR was computed for each echo time (TE) separately and for their combination as described inKundu et al. (2017), by computing the T_2_^*^at each voxel and combining the echoes’ data with the following weights:wecho=TEechoe−TEechoT2*∑TEechoe−TEechoT2*(echo here stands for the echo number).

In addition to the ETR, the CNR at each voxel and scan was evaluated. The CNR was defined as:CNR=$\left({\langle \Delta S_{v,inorm}\text{​}\left(\tau \right)\rangle }_{ONwindow}\text{​}-\;\text{​}{\langle \Delta S_{v,inorm}\text{​}\left(\tau \right)\rangle }_{OFFwindow}\right)/\text{​}\langle \sigma_{S}\text{​}\left(\tau \right)\rangle$., where ONwindow and OFFwindow are the same time windows as above. Since the rotation resulted in different signal changes in different voxels (as shown inFig. 1d), the set of voxels in each experiment delivered a range of CNR values, thus providing an additional dimensionality in one experiment.

It is important to note that in many phantoms, the SNR can be much higher than in*in-vivo*scans, in which cases it will not provide a representative ETR. To achieve a tSNR similar to realistic in-vivo scans, the flip angle of the scans in the phantom was scaled respectively. SeeFigure 2bfor the phantom versus in-vivo tSNR comparison.

### 2.5 Examining the ETR dependencies

Five experiments were executed to examine the effects of the scan parameters, block design, and tissue properties on the ETR. The TR of the scans in all experiments except the one that examined TR dependence was 600 ms. The first experiment was performed to investigate the ETR-dependence on tissue properties, comparing short and long T_2_^*^phantoms. Another experiment examined the dependence of ETR on TR, which was performed with the phantom mimicking the cortex T_2_^*^. Motivated to optimize the ETR for the BG region, several experiments were performed with the short T_2_^*^phantom, where the effects of voxel size on the stimulus paradigm design were investigated. A detailed description of each experiment is summarized below.

#### 2.5.1 Experiment 1: ETR as a function of T _2_ *

To examine ETR as a function of T_2_^*^, 6 repeated scans with each of three ∆t (0, 60, & 600 ms) were performed, resulting in a total of 18 scans for each agarose composition. The scan included six blocks of “ON”/”OFF” states, accumulating to a total scan time of 258 seconds. The ETR of each echo of a multi-echo EPI and their combination was calculated and averaged for the three onset times. To plot CNR dependence, the voxels were divided into bins according to their CNR, and ETR was computed for each bin.

#### 2.5.2 Experiment 2: ETR as a function of voxel size

To examine the dependence of ETR on voxel size, three voxel sizes were imaged using the short-T_2_^*^phantom: (i) 2.1 × 2.1 × 3 mm^3^, (ii) 1.5 × 1.5 × 3 mm^3^(2-fold smaller volume), and (iii) 1.5 × 1.5 × 1.5 mm^3^(4-fold smaller volume compared to the first set). Each voxel size was scanned seven times (each with six blocks of the same paradigm as in Experiment 1–3 scans with ∆t = 0 ms, 2 scans with ∆t = 60 ms, and 2 scans with ∆t = 600 ms). To increase the number of voxels in this analysis, the t-test threshold was reduced to 4 in the 1.5 × 1.5 × 3 mm^3^and the 1.5 × 1.5 × 1.5 mm^3^datasets.

#### 2.5.3 Experiment 3: ETR as a function of TR

In this experiment, eight scans were performed with the same paradigm as in Experiment 1 with ∆t = 0 on the long-T_2_^*^phantom for each of five different TRs: 600, 800, 1000, 1500, and 2000 ms. The ETR as a function of the TR and the CNR were estimated for the combined echoes’ data.

#### 2.5.4 Experiment 4: ETR as a function of the experimental design

A set of experiments was designed to explore different lengths of the block’s “ON” state and different block densities, while keeping the same (or similar) total scan time. Block’s T_ON_/T_OFF_/N_cycles_duration in this experiment was set to 15/18/6,12/18/7,9/18/8, 6/18/9, 9/13/9, and 6/10/12 seconds. This set of scans was performed with the long-T_2_^*^phantom.

An experiment with short stimuli was also performed, to examine CNR-levels similar to event-related paradigm. This experiment included 20 events of 1 second stimuli with 10 seconds between the events. Twelve repeated scans were performed to estimate the ETR dependence in number of events.

#### 2.5.5 Experiment 5: ETR dependence on the shape of the HRF and on the physiological noise

Two experiments were conducted to examine how the ETR is affected due to variation in the evoked responses that have neurovascular or physiological origin. The first was performed with varying undershoot of the HRF and the second with extra, randomly shifted, signal fluctuations at the respiratory frequency.

To examine the effect of the HRF shape on the ETR, the rotation angles’ sequence was designed with three levels of the HRF undershoot. The HRF was produced using SPM software (“SPM Toolkit,” 1997), where the relative magnitude of the undershoot can be controlled. The default parameter in the SPM package for undershoot (p(5) = 6) and two additional levels (p(5) = 4 and p(5) = 9) were used—multiplying the default undershoot ratio by ×0.67 and ×1.5. In this experiment, six scans for each undershoot were performed with the same paradigm as in Experiment 1. A separate analysis for each undershoot was performed to estimate the ETR for each case. To examine the effect of the varying undershoot, the ETR based on time delays over all shapes was estimated (the delays were compared to a reference scan with the default undershoot).

To examine the effect on the ETR due to breathing-induced signal fluctuations, the rotation sequence was designed to include signal fluctuations with a sine function at 0.2 Hz frequency and a relative magnitude of ×0.2 compared to the HRF response. This signal was added to the rotation angles’ sequence with a different random time shift each time. In this experiment, six scans with added physiological noise and six scans without it were performed with the same paradigm as in Experiment 1. A separate analysis for each set was performed to estimate the ETR.

#### 2.5.6 Experiment 6: Validation of the time delay estimation and its accuracy

To examine our capability to estimate time delays shorter than the TR, 10 scans were repeated. Each scan included eight blocks with six onset times (Δt = 0, 60, 180, 300, 420, 540 ms). Δt = 0 ms was repeated in three blocks, with the first one serving as a reference. The estimated time delay for each block was calculated by averaging the$\Delta S_{v,inorm}\left(\tau \right)$of each block and calculating the*t_est_*for each voxel compared to the reference. This set of scans was performed with the short-T_2_^*^phantom.

A table summarizing the number of voxels included in the analysis to estimate the ETR in each experiment is included as Supporting InformationTable S1.

### 2.6 MRI scanning

All scans in this study were performed on a 7 T MRI system (MAGNETOM Terra, Siemens Healthcare, Erlangen) using a commercial 1Tx/32Rx head coil (Nova Medical, Wilmington, MA). A multi-echo EPI pulse sequence from CMRR (Center for Magnetic Resonance Research, Minnesota, USA) was used (Feinberg et al., 2010;Moeller et al., 2010). The following common scan parameters were employed: TR = 600 ms (except in Experiment 3, as described in the dedicated section), voxel size = 2.1 × 2.1 × 3 mm^3^(except in Experiment 2), 6 slices, flip angle = 10°, and reference amplitude in the range of 22–26 V (set by dividing the adjustment reference amplitude by 5). The flip angle and reference amplitudes were set to achieve a similar tSNR to that measured*in vivo*. Echo spacing of 0.53 ms (with a bandwidth per pixel of 2480 Hz) was used in most of the experiments. The scans in Experiment 4 part 2 and Experiment 5 were performed with an echo spacing of 0.6 ms (with a bandwidth per pixel of 1860 Hz). The echo spacing was changed (from 0.53 ms to 0.6 ms) to shift the gradient switching frequency away from possible acoustic resonances of the system and thus better safeguard the gradients.

The specific scan parameters in experiments 1, 2, and 4 (part 1) were as follows: For the short-T_2_^*^phantom set, TEs of 7.68, 19.42 & 31.16 ms, FOV = 171 × 240 mm^2^, matrix size = 80 × 112, and acceleration factor 3 using GRAPPA. For the long T_2_^*^phantom, TEs of 14.4, 43.63 & 72.86 ms, FOV = 153 × 238 mm^2^, matrix size = 72 × 112, and no acceleration.

The specific scan parameters in Experiment 2 were as follows: For voxels size (i), TEs of 11.60, 30.92, & 50.24 ms (FOV = 171 × 240 mm^2^, matrix size = 114 × 160, bandwidth per pixel 1955 Hz). For voxel size (ii), TEs of 11, 30.32, & 49.64 ms (FOV = 171 × 240 mm^2^, matrix size = 114 × 160, bandwidth per pixel 1955 Hz). For voxel size (iii), TEs of 7.68, 19.42, & 31.16 ms.

The specific scan parameters in Experiment 4 (part 2) and Experiment 5 were TEs of 15.4, 46.12, & 76.84 ms, FOV = 140 × 238 mm^2^, matrix size = 66 × 112, and no acceleration.

## 3 Results

**Fig. 4.**
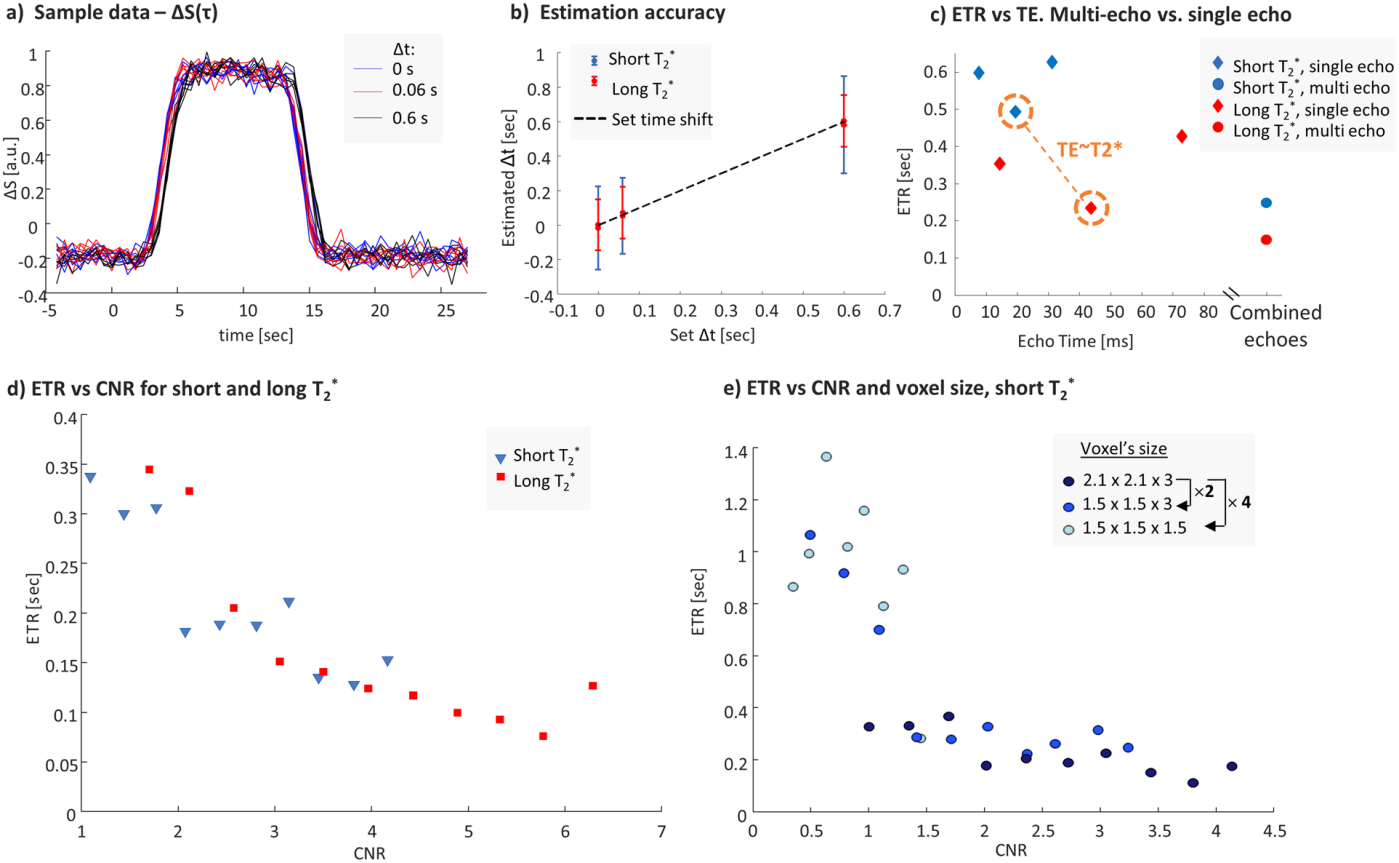
ETR dependence on tissue and scan parameters, including the T_2_^*^, TE, CNR, and voxel size. (a) Signal change for three Δt in a sampled voxel in Experiment 1. (b) Estimated Δt in the long-T2^*^phantom and the short-T2^*^phantom (Experiment 1). (c) ETR versus TE versus combined echoes for the short- and long-T_2_* configurations. (d) ETR as a function of the CNR for the short- and long-T_2_^*^configurations. (e) ETR as a function of the CNR for three different voxel sizes.

To examine ETR as a function of T_2_^*^(Experiment 1), six scans with three different onsets were performed for each agarose composition.Figure 4ashows a sample set of the repeated scans. The ETR, defined as the standard deviation of the time shifts over repeated scans, was calculated for each echo and for combined echoes and then averaged over the three onset times (Fig. 4c). In this comparison, the ETRs of all voxels for each TE were averaged. The shortest ETR was at TE≈T_2_^*^- 243.1 ms and 493.3 ms for the long T_2*_and short T_2_^*^, respectively. This result is expected, as previous works showed that maximal CNR is achieved at TE = T_2_^*^. Combining the echoes’ data, the ETR was 150.8 ms and 248.2 ms for the long- and short-T_2_^*^phantom, respectively, which is 1.6-fold and 2-fold lower than for a single echo. The estimated time shifts for the three onset times, ∆t (0, 60, & 600 ms) averaged over six repeated scans, were 1.6 ms, 71.7 ms, and 603 ms for the long-T_2_^*^phantom and -17.4 ms, 53.1 ms, and 580.35 ms for the short-T_2_^*^phantom (Fig. 4b).Figure 4dshows the dependence of the ETR on the CNR (combined echoes), with an evident reduction in the ETR as the CNR increases. Histograms of the estimated time shifts for all voxels and scans in experiments with the long- and short-T_2_^*^phantom are shown in the Supporting InformationFigure S2 and S3. In these histograms, the short-T_2_^*^phantom, having lower CNR than in the long-T_2_^*^phantom, includes several outliers. These outliers belong to voxels at the ends (along the radial dimension) of the border between the compartments of the phantom, voxels which were identified as prone to larger errors. These outliers slightly shifted the average time shifts.

The ETR’s dependence on the voxel size is indicated by a decrease in the CNR and an increase in the ETR for smaller voxel sizes (Experiment 2;Fig. 4e). The mean and SD of the ETR calculated using the CNR bins for a volume of 2.1 × 2.1 × 3 mm^3^, half that volume, and a quarter of that volume were 227 ± 86 ms, 428 ± 259 ms, and 927 ± 316, respectively. Note that for half the volume, the ETR was 1.6 times longer, and for a quarter of the volume, it was 3 times longer.

**Fig. 5.**
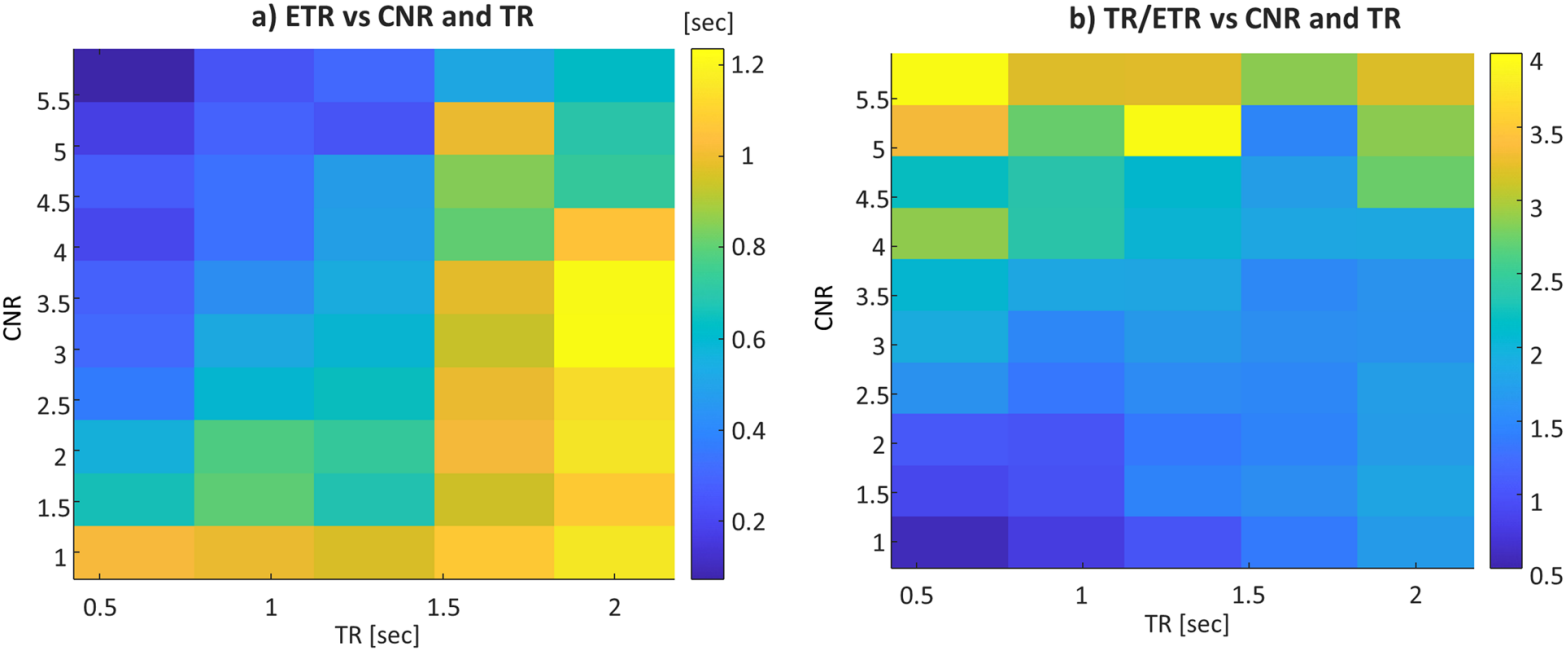
ETR dependence on TR. (a) ETR as a function of the CNR and TR. (b) TR∕ETR (or the reduction factor in the actual time resolution) as a function of the CNR and TR.

The TR and CNR were also found to contribute to the ETR (Experiment 3;Fig. 5). For example, for a TR of 600 ms, the ETR is in the range of 72–1000 ms, whereas for a TR of 2000 ms, the ETR is in the range of 700–1200 ms, depending on the CNR. This highlights the vital importance of an attainable CNR.

**Fig. 6.**
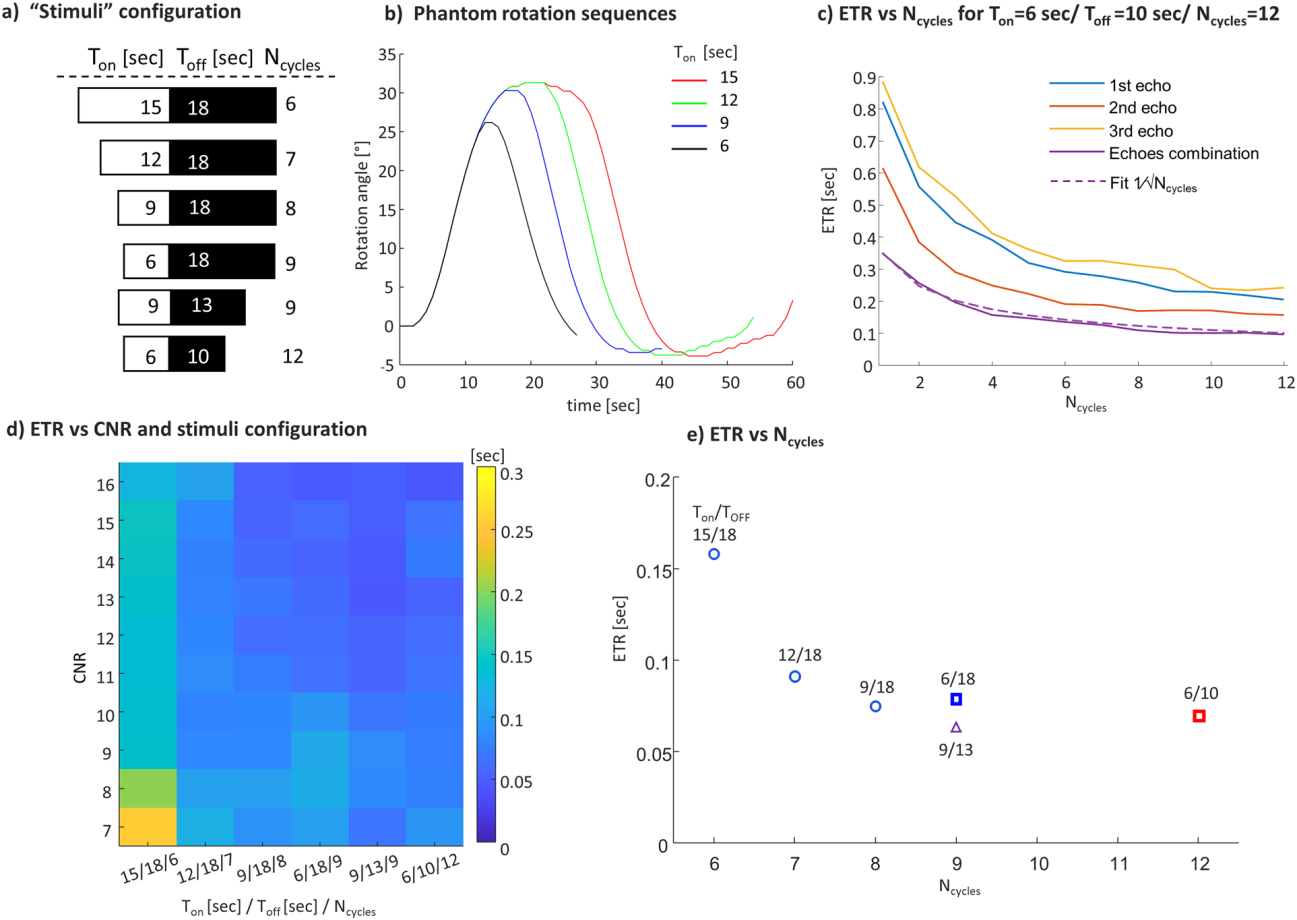
ETR as a function of the blocks’ number and density. (a) The examined “stimuli” configuration. (b) Phantom rotation sequences for different paradigms. (c) ETR as a function of N_cycles_calculated for the experiment with T_ON_= 6 seconds, T_OFF_= 10 seconds, and N_cycles_= 12. (d) ETR as a function of the CNR and the paradigm configuration. (e) ETR as a function of N_cycles_.

We also inspected the influence of the block design parameters (Experiment 4). First, the ETR as a function of the number of blocks was tested based on a repeated set of scans with T_on_= 6 seconds, T_OFF_= 10 seconds, and N_cycles_= 12. The results show that the ETR improves as the number of blocks increases, having an approximate dependence of √N_cycles_(Fig. 6c).Figure 6also shows the ETR for single- and multi-echo datasets, with the shortest ETR using combined echoes. Second, the effect of the number and the length of blocks in a scan were examined, while preserving the length of the total scan time. We compared 6 paradigms, 4 of which had a fixed T_OFF_of 18 seconds and a varying T_ON_(6, 9, 12, and 15 seconds) and 2 of which had a shorter T_OFF_(Fig. 6d–e).Figure 6dshows the dependence of the ETR on the CNR for each paradigm.Figure 6eshows the average ETR for each paradigm. A clear reduction in ETR is observed as the number of blocks increases. However, the paradigm with T_on_= 6 seconds has slightly higher values compared to that with T_on_= 9 seconds. This can be explained by the phantom’s slightly lower rotation angles generating the HRF response for T_ON_= 6 seconds; thus, the response function is shorter (seeFig. 6b), which means less meaningful signal to correlate.

**Fig. 7.**
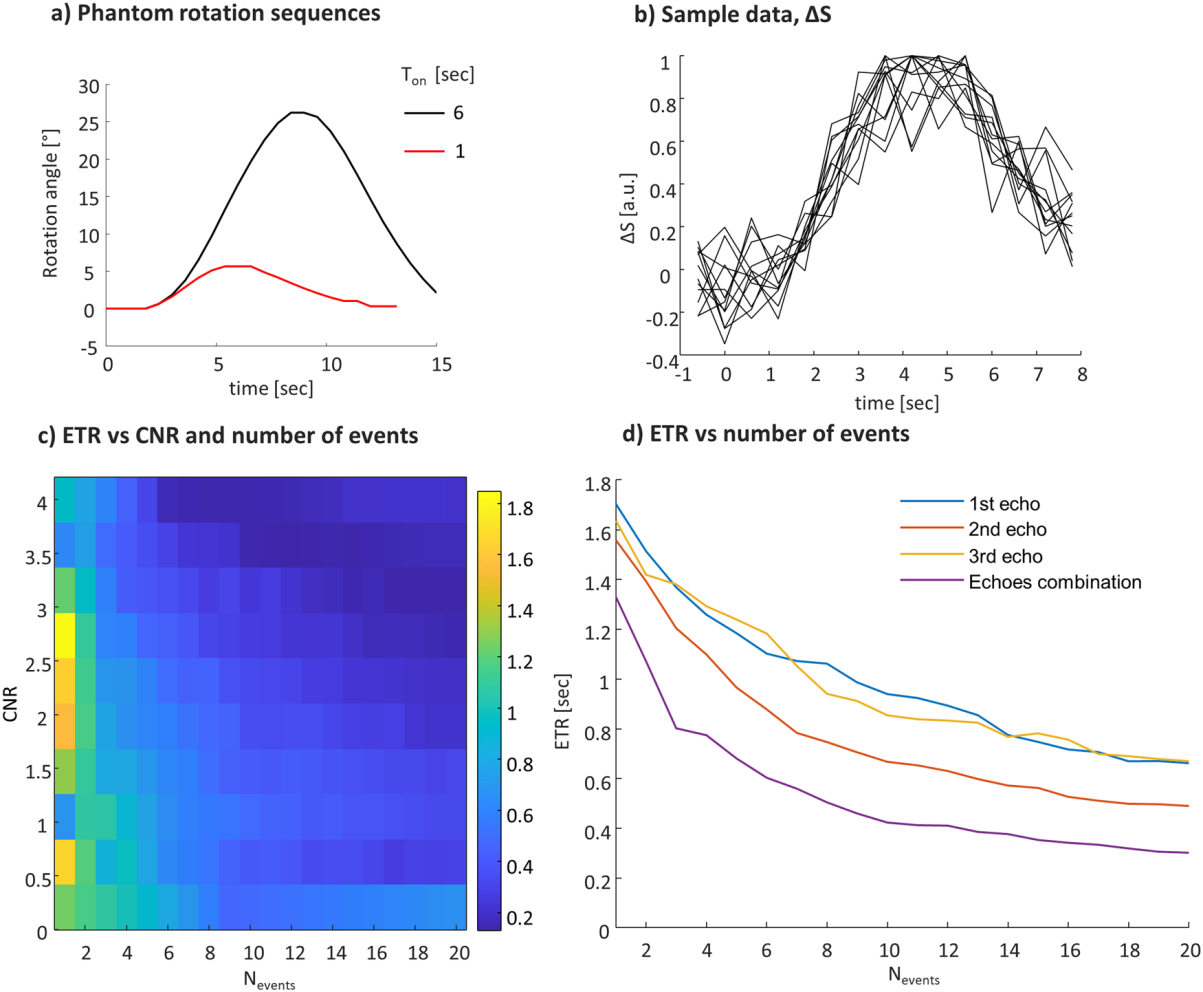
ETR in a short-stimuli experiment. (a) Rotation sequence for short-stimuli compared to 6-seconds stimuli (both with 10 seconds between the stimuli). (b) Signal change in a sampled voxel. (c) ETR as a function of the CNR and the number of events. (d) ETR as a function of the number of events for single- and multi-echo analysis.

Figure 7shows the results of the experiment designed with repeated short-stimuli events that mimic the CNR of the event-related paradigm. In this case, the achieved CNR is much lower, as expected for short-stimuli, and therefore the ETR is prolonged compared to block-design experiments. However, the ETR improves when increasing the number of events.

**Fig. 8.**
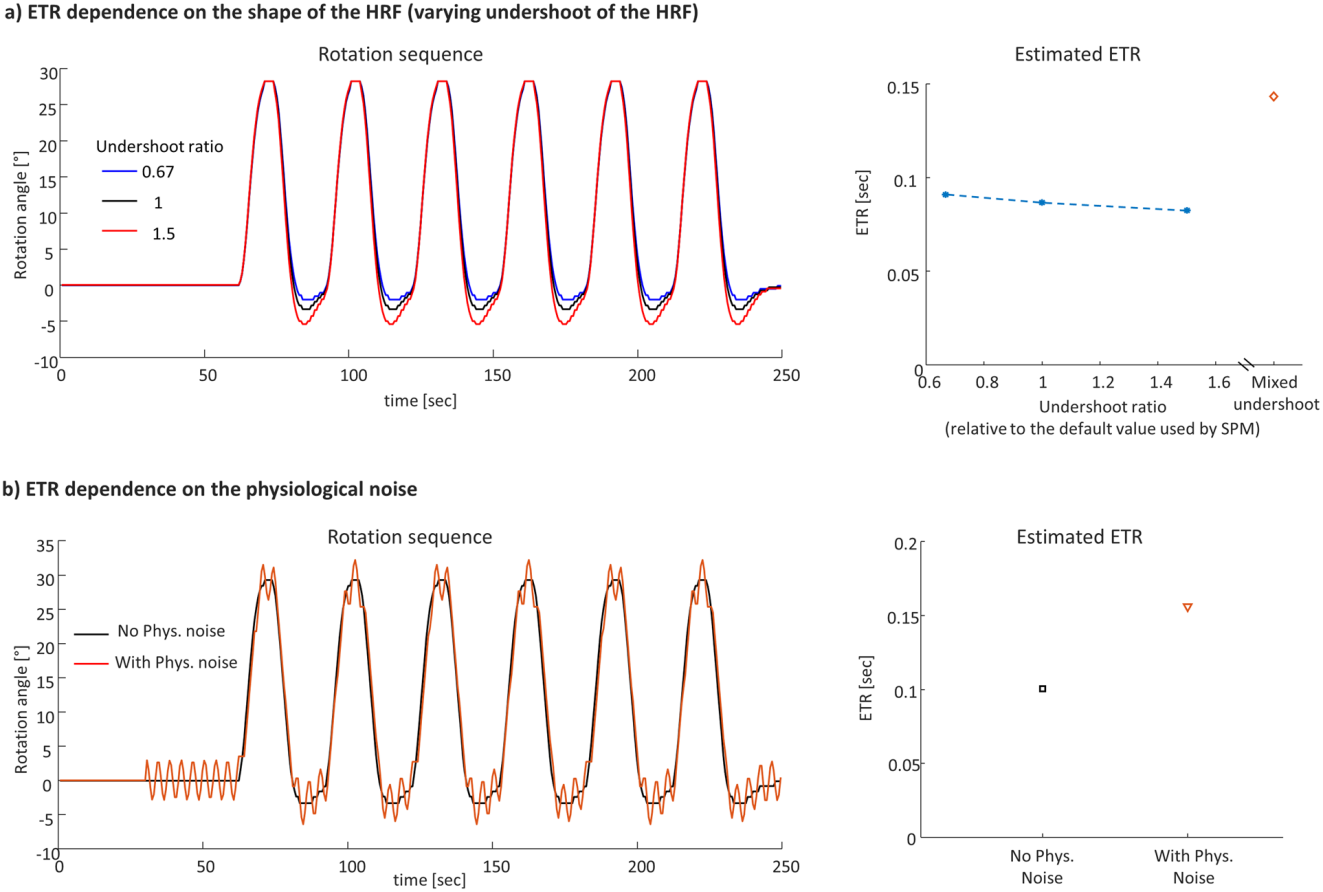
ETR dependence on the shape of the HRF and on the physiological noise. (a) ETR dependence on the shape of the HRF (varying undershoot of the HRF). Left: Rotation sequence for three undershoot levels, right: the ETR as function of the undershoot ratio and ETR for mixed analysis. (b) ETR dependence on the physiological noise. Left: Rotation sequence with and without added physiological noise, right: the estimated ETR.

Figure 8ashows the ETR dependence on the magnitude of the HRF undershoot. Analyzing the datasets of each undershoot independently, the ETR slightly improved with increase of the undershoot. This can be expected, since with a larger undershoot, we expect more meaningful signal to correlate. Mixing the measurements of different undershoots prolongs the ETR, since the variation in the undershoot is interpreted as a bias in the estimated time delay.Figure 8bshows the ETR degradation in measurements with added fluctuations mimicking physiological noise due to respiration.

**Fig. 9.**
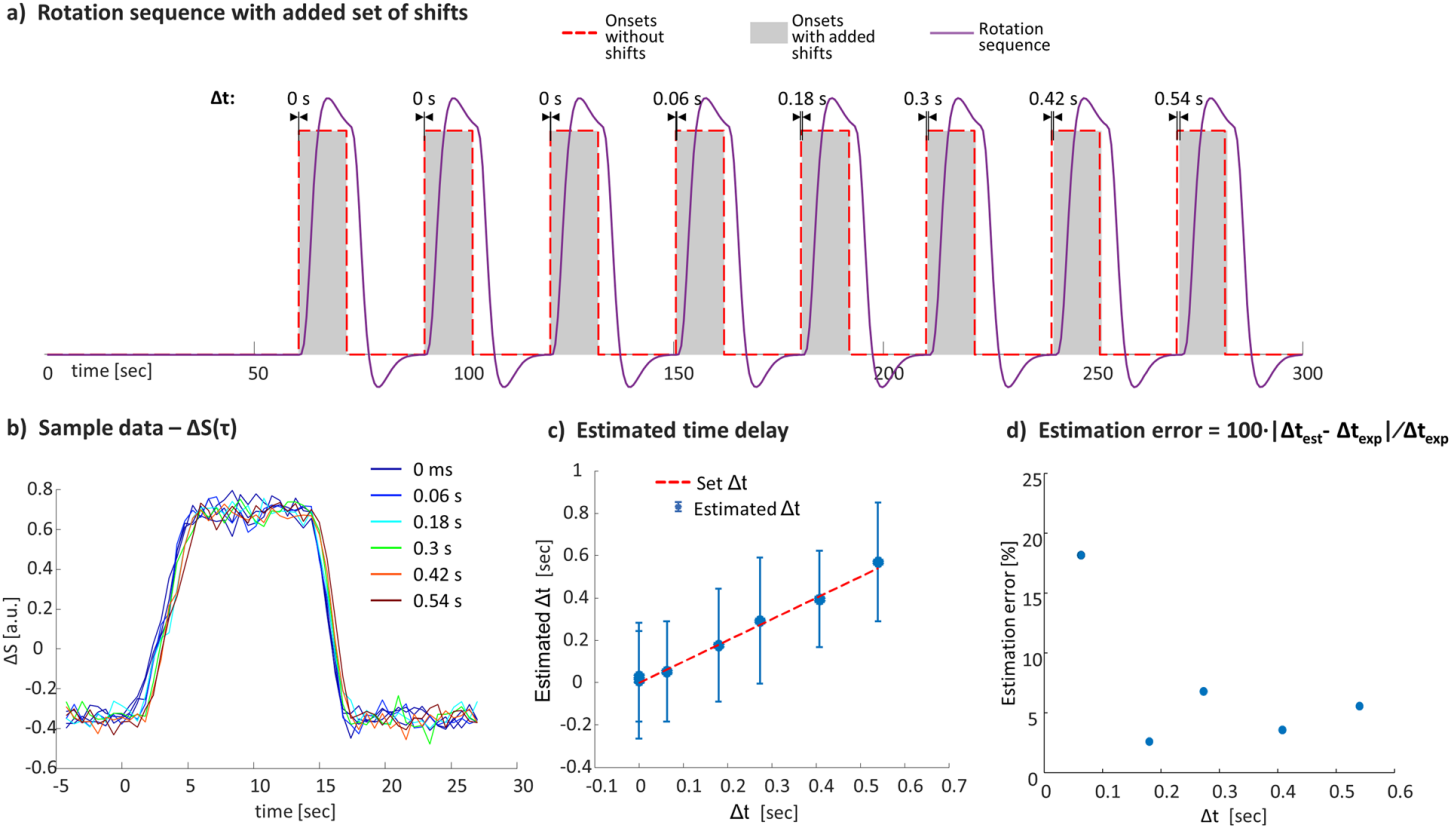
Accuracy of the estimated time delay. (a) A rotation sequence that included blocks with shifted onset times. (b) Signal change in a sampled voxel for different set Δt. (c) Estimated time delays as a function of the expected Δt. (d) Estimated relative error of the time delay as a function of the set Δt.

To examine the accuracy of the time delay estimation, separate blocks from 10 repeated scans were averaged to estimate the t_est_compared to the reference (Experiment 6;Fig. 9a).Figure 9bshows the signal change from a sample voxel for the different blocks.Figure 9cand9dshow the estimated time delay and the relative error, respectively. The results show a 4.6 ± 1.0% error for the Δt > 60 ms, versus an 18% error for Δt = 60 ms.

## 4 Discussion and Conclusions

In this study, the effective temporal resolution (ETR) in a task-based fMRI experiment was investigated using a dynamic phantom. The dynamic phantom was programmed to generate rotation sequences that produce MRI signals that mimic a block-design fMRI. A set of experiments was performed to study the ETR dependence on different parameters and to verify that different onset times of the blocks can be correctly assessed. The estimated ETR here represents the*minimal time shift difference*we can experimentally discern due to the noise in the system. The first set of experiments examined the possible ETR in an fMRI experiment using an EPI scan with a typical spatial resolution (2.1 × 2.1 × 3 mm^3^) and a TR of 600 ms. The ETRs were evaluated for single-echo versus multi-echo and in two phantoms that mimic T_2_^*^of cortex-like and BG-like environments. When comparing the signals arising from long- and short-T_2_^*^regions, a substantially lower ETR was observed in the latter. Thus, studying the timing of sub-cortical signals that have shorter T_2_^*^values, like the basal ganglia, is expected to be more challenging compared to ones with longer T_2_^*^values, like the cortical regions. Combining the data from all the echoes can improve the ETR, as indicated by our results showing a shorter ETR, by a factor of at least 1.6 times, when combining echoes compared to a single-echo. Note, that single-echo offers shorter acquisition compared to a multi-echo, therefore it can be planned with shorter TR and thus improve the ETR. However, the benefit of multi-echo is that it can provide optimal TE’s for multiple regions in the brain with range of T_2_^*^. These tradeoffs should be considered in the design of a particular experiment.

Notably, the ETR (computed over all voxels) for a single-echo EPI was 2.5× and 1.2× shorter than the TR for the cortex-like and BG-like tissues, respectively. When combining the echoes, the ETR was 4× and 2.4× shorter than the TR. Note, that the ETR was defined here as one standard deviation of a set of repeated measurements, which represents 68% of observed data. If higher confidence is required, two or three standard deviations should be used (representing 95% and 99% of observed data). Still, an ETR that is much shorter than TR is feasible, since the statistical power of the experiment combines all data points to estimate the time delay.Figure 5shows the ETR dependence on CNR and TR, where with a very high CNR the ETR can reach even 72 ms while still scanning with a TR of 600 ms. However, a substantial penalty will be paid if the CNR is low, in which case the ETR can be larger than the set’s TR. Therefore, to study timing responses, it is imperative to estimate the potential ETR by taking into account the planned scan parameters and experimental paradigm. Note, that the shortest TR available with the dynamic phantom was 600 ms. However, it allowed us to study range of parameters, providing useful insights for ETR optimization.

Our results demonstrate an expected tradeoff between the spatial and temporal resolution. Namely, scanning at higher spatial resolutions can result in lower temporal resolution. This should be taken into consideration in studies aiming to investigate the BOLD signal arising from small-scale brain regions, such as the cortical laminae or brainstem nuclei. Although such spatial scales are possible, especially at 7 T MRI, the restricted CNR still hinders a fine temporal characterization of the measured signal. Studies that aim to achieve both high spatial and high temporal resolutions should carefully consider the experimental parameters, the subsequent CNR, and the statistical power.

Our assessment of the effect of the functional paradigm—the blocks’ duration and density—on the ETR indicates that, as long as the CNR is kept constant, the ETR can be shortened by increasing the number of block-cycles in the experiment (seeFig. 6e). However, increasing the density of the blocks eventually comes at the expense of the CNR, when the stimuli duration, evoking the signal rise, is reduced.

Furthermore, we performed a feasibility analysis showing that the dynamic phantom can be used to study the ETR performance under varying HRF shapes and physiological noise. Varying the HRF undershoot magnitude through the experiment as well as adding fluctuations mimicking physiological noise due to breathing will result in longer ETR. A further study that examines filtering the physiological noise or using ICA analysis to remove the physiological noise could improve the resulting ETR.

We also verified that the predefined time delays of onsets much shorter than the TR can be accurately assessed. Our results (Experiment 6) of a set of scans with different time delays show high accuracy in the estimated time shifts—with a <7% relative error for Δt > 60 ms and an 18% error for Δt = 60 ms.

Taken together, our results show the great prospect of studying timing responses with fMRI, by demonstrating that a very short ETR can be achieved. It is the long HRF, of several seconds, that allows us to reach a short ETR, as it includes many points we can use for the cross correlation. While a simple strategy of cross-correlation between two signals was used to estimate the time-delay in this study, implementing more sophisticated analyses, such as adding additional fitting properties, can further improve the attainable ETR.

Although the dynamic phantom permits a straightforward assessment of the ETR, it has certain shortcomings. In the current implementation, only a simple physiological noise model was included in the analysis of the ETR, while a real*in-vivo*fMRI would include additional factors, such as faster fluctuations due to cardiac frequency and movement-related noise. Further studies aimed at improving the simulation of a realistic experiment are required. Other tissue properties and different functional paradigms can also be designed to study the ETR. In future studies, advancements in the current setup might allow further investigation of fMRI’s ETR. Since many studies aim to probe timing parameters with very short TRs (<100 ms), it would be insightful to examine their resulting ETR.

## Supplementary Material

Supplementary Material

## Data Availability

The code and data will be made available via a request to the corresponding author.
