# Supplementary material for: Investigating the effective temporal resolution in a task-based functional MRI experiment at 7 T MRI using a dynamic phantom
Source: Imaging Neurosci (Camb). 2024 Oct 10;2:imag-2-00309. doi: 10.1162/imag_a_00309 (PMC12290576; doi:10.1162/imag_a_00309)
Supplement: Supplementary Material [file imag_a_00309-supp.pdf]

Supporting Information:

Investigating the effective temporal resolution in a task-based functional MRI experiment at 7 T MRI using a dynamic phantom

Guy Baz[1,2], Rita Schmidt[1,2]

[1]Weizmann Institute of Science, Department of Brain Sciences, Rehovot, Israel

[2]The Azrieli National Institute for Human Brain Imaging and Research, Weizmann Institute of Science

S1. Simulation of two evoked responses to stimuli onsets with small time-delay

In this study we aim to investigate what is the effective temporal resolution (ETR) that a set of chosen scan parameters and an fMRI paradigm can supply. This time resolution represents the minimal time delay that can be discerned between evoked responses with high enough statistical confidence. CNR of the measured signal changes can affect the ETR, even when the TR is kept constant. To demonstrate it, we performed a simulation of the expected evoked responses from each of two stimuli, where the onset of the two, relative to the scan, differed by 200 ms. The signal responses were simulated with three CNR levels with the same TR of 600 ms. The three levels– low, mid and high – were chosen such that the estimated ETR was, respectively, much longer than the time delay difference, close to the time delay difference and much shorter than the time delay difference. Figure S1 shows the simulated signals. In the high CNR case the difference in magnitude between two evoked signals is clearly seen even with 200 ms delay, while the signal in the low CNR case is too noisy to make this distinction.

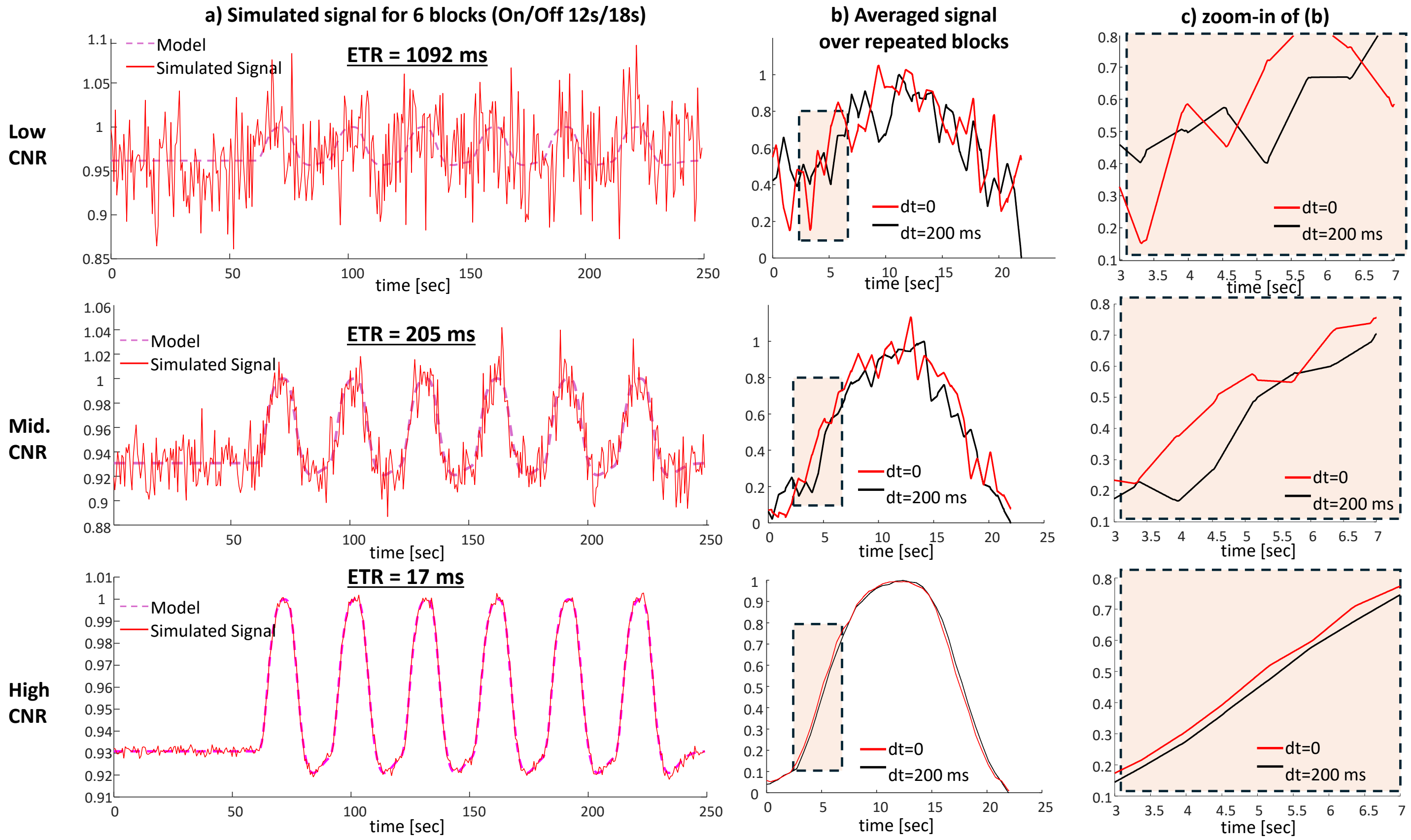

Figure S1: Simulation of two evoked signals, from two stimuli with a 200 ms onset time difference, at three CNR levels (low, mid and high). The figures from left to right: a) simulated signal for 6 stimuli blocks, b) the two time offset (200 ms) signals averaged over the blocks, c) zoom ins of (b) during the rise time. The ETR for each case was estimated from 100 repeated simulated scans.

S2. Histograms of the estimated time delays for experiment #1

To examine the ETR as a function of T_2^*, 6 repeated scans, with each of three Δt (0, 60 & 600 ms) were performed, resulting in a total of 18 scans for each agarose composition (long T_2^* and short T_2^*). Figure S2 shows the histograms of the estimated time delays with the long T_2^* setup. Figure S3 shows the resulting histograms for the short T_2^* setup. In the latter setup, due to lower CNR, several outliers that slightly shift the average estimated Δt were observed. These outliers were voxels that were located at or very close to the center of the cylinder axis (thus behaving more erratically, because it does not rotate too much) or voxels at the edge of the phantom, where the B_0 inhomogeneity could cause higher errors. Removing the outliers brings the average values close to the expected time-delay. Figure S3 shows the histograms with the outliers and without them.

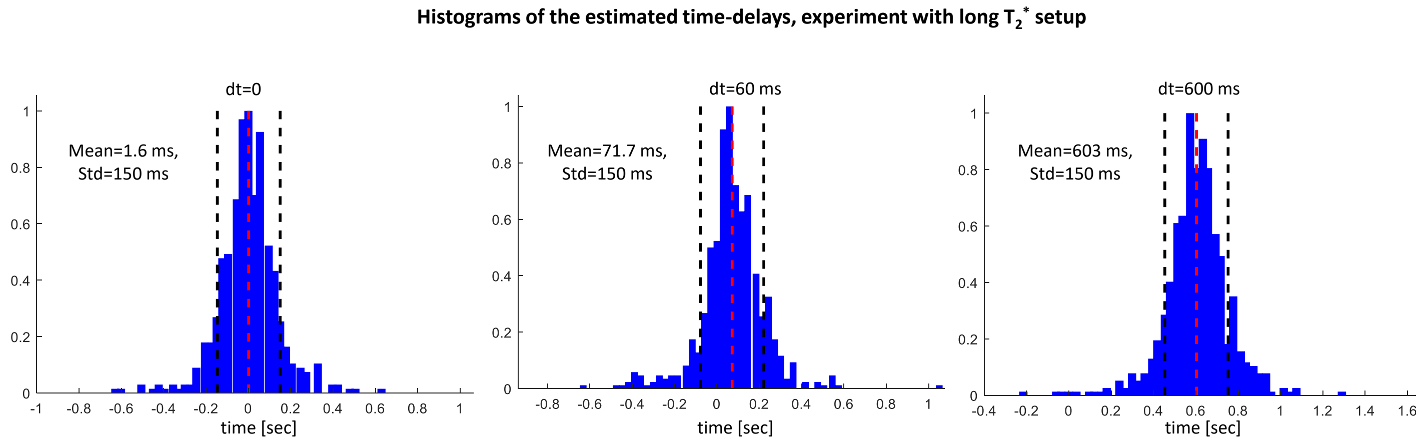

Figure S2: Histograms of the estimated time delays with the long T_2^* setup for Δt=0, Δt=60ms and Δt=600 ms.

a) Histograms of the estimated time-delays , before removing outliers

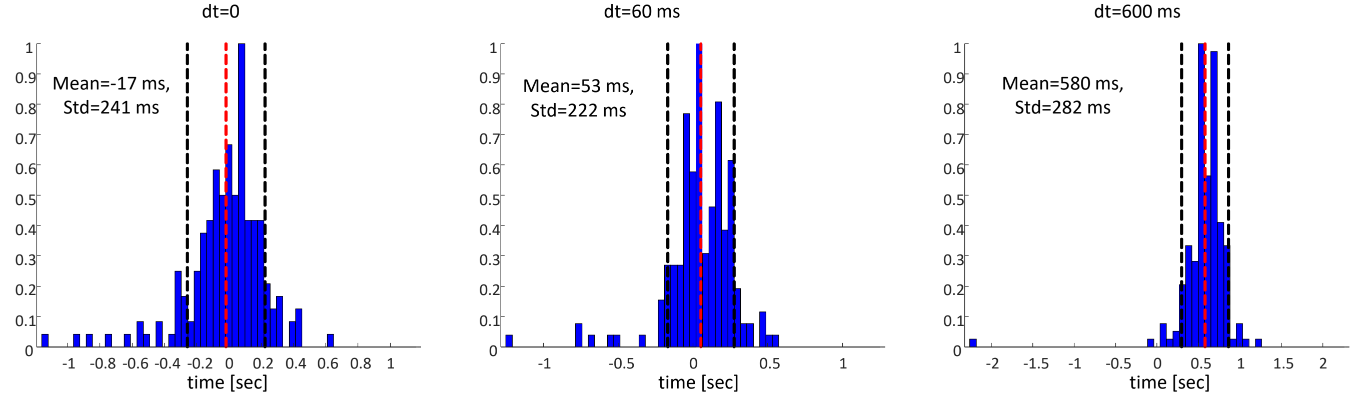

b) Histograms of the estimated time-delays , after removing outliers

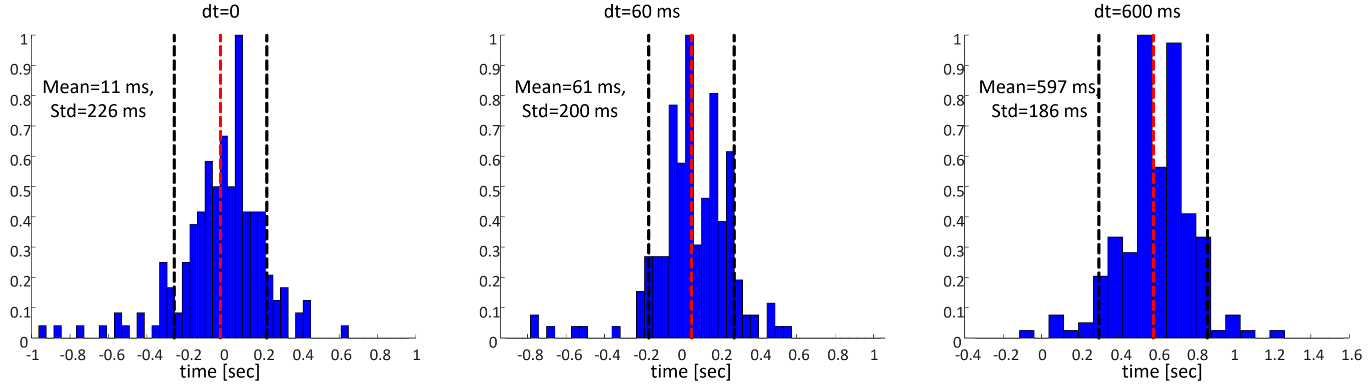

Figure S3: Histograms of the estimated time delays with the short T_2^* setup for Δt=0, Δt=60ms and Δt=600 ms. a) histograms including the outliers, b) histograms with the outliers removed.

Table S1. Summary of the experiments' info

Experiment	Condition	Number of repetitions/scans	The phantom being used	T-test threshold	Number of voxels
1. T2*	Short T2*	18	short and long T2*	8	87
	Long T2*			10	111
2. Voxel's size	1.5 X 1.5 X 3	7	Short T2*	4	116
	1.5 X 1.5 X 1.5			4	49
3. TR	600	8	Long T2*	10	91
	800				
	1000				
	1500				
	2000				
4a. Number and block's length	15/18/6	6	Long T2*	10	129
	12/18/7				137
	9/18/8				114
	6/18/9				111
	9/13/9				120
	6/10/12				71
4b. Short-stimuli to mimic event-related fMRI	-	20	Long T2*	5	84
5a. manipulating the HRF undershoot	small	6	Long T2*	10	89
	regular	6			
	large	6*			
5b. physiological noise	With noise	6	Long T2*	10	121
	without	6			
6. Validation of the time delay estimation and its accuracy		10	Short T2*	8	105

* One of the scans in this set was removed from the analysis due to an error in the synchronization between the rotation sequence and the MRI scan.
